# Investigating brain cortical activity in patients with post-COVID-19 brain fog

**DOI:** 10.3389/fnins.2023.1019778

**Published:** 2023-02-09

**Authors:** Grzegorz M. Wojcik, Oren Shriki, Lukasz Kwasniewicz, Andrzej Kawiak, Yarden Ben-Horin, Sagi Furman, Krzysztof Wróbel, Bernadetta Bartosik, Ewelina Panas

**Affiliations:** ^1^Department of Neuroinformatics and Biomedical Engineering, Institute of Computer Science, Maria Curie-Sklodowska University in Lublin, Lublin, Poland; ^2^Department of Cognitive and Brain Sciences, Ben-Gurion University of the Negev, Beer-Sheva, Israel; ^3^Department of Computer Science, Ben-Gurion University of the Negev, Beer-Sheva, Israel; ^4^Department of International Relations, Faculty of Political Science and Journalism, Maria Curie-Sklodowska University in Lublin, Lublin, Poland

**Keywords:** brain fog, EEG, ERP, COVID-19, cortical activity, LDA

## Abstract

Brain fog is a kind of mental problem, similar to chronic fatigue syndrome, and appears about 3 months after the infection with COVID-19 and lasts up to 9 months. The maximum magnitude of the third wave of COVID-19 in Poland was in April 2021. The research referred here aimed at carrying out the investigation comprising the electrophysiological analysis of the patients who suffered from COVID-19 and had symptoms of brain fog (sub-cohort A), suffered from COVID-19 and did not have symptoms of brain fog (sub-cohort B), and the control group that had no COVID-19 and no symptoms (sub-cohort C). The aim of this article was to examine whether there are differences in the brain cortical activity of these three sub-cohorts and, if possible differentiate and classify them using the machine-learning tools. he dense array electroencephalographic amplifier with 256 electrodes was used for recordings. The event-related potentials were chosen as we expected to find the differences in the patients' responses to three different mental tasks arranged in the experiments commonly known in experimental psychology: face recognition, digit span, and task switching. These potentials were plotted for all three patients' sub-cohorts and all three experiments. The cross-correlation method was used to find differences, and, in fact, such differences manifested themselves in the shape of event-related potentials on the cognitive electrodes. The discussion of such differences will be presented; however, an explanation of such differences would require the recruitment of a much larger cohort. In the classification problem, the avalanche analysis for feature extractions from the resting state signal and linear discriminant analysis for classification were used. The differences between sub-cohorts in such signals were expected to be found. Machine-learning tools were used, as finding the differences with eyes seemed impossible. Indeed, the A&B vs. C, B&C vs. A, A vs. B, A vs. C, and B vs. C classification tasks were performed, and the efficiency of around 60–70% was achieved. In future, probably there will be pandemics again due to the imbalance in the natural environment, resulting in the decreasing number of species, temperature increase, and climate change-generated migrations. The research can help to predict brain fog after the COVID-19 recovery and prepare the patients for better convalescence. Shortening the time of brain fog recovery will be beneficial not only for the patients but also for social conditions.

## 1. Introduction

The COVID-19 pandemic has changed the functioning of whole societies and post-pandemic economic and social problems are going to manifest themselves probably for many years when it is over (Ciotti et al., 2020). The most common symptoms of COVID-19 comprise cough, fever, breathing problems, and fatigue. Nevertheless, there is a wide range of neurological symptoms associated with COVID-19 in up to 25% of those who survived the disease. One of the most often reported is the so-called brain fog (Ocon, 2013; Kovalchuk and Kolb, 2017; Kverno, 2021; Asadi-Pooya et al., 2022; Callan et al., 2022; Hugon et al., 2022; Krishnan et al., 2022). It appears 2–3 months after the infection and can last up to 6 months or sometimes longer. It is indexed neither in ICD-10 nor in DSM-V; however, the symptoms resemble some of those characteristics of CFS (chronic fatigue syndrome) (Afari and Buchwald, 2003), which are diagnosed as follows Chen (1986), Kroenke et al. (1988), David et al. (1990), Bates et al. (1993), and Pawlikowska et al. (1994):

- Memory problems.
- Inability to focus or concentrate.
- Difficulty in processing information.
- Trouble in problem-solving.
- Feelings of confusion or disorientation.
- Having a hard time while calculating.
- Diminished visual and spatial skills.
- Trouble in finding proper words.
- Trouble in recognizing known faces.

As Ross mentions in JAMA (Ross et al., 2004), chronic fatigue syndrome (CFS) is a challenge faced by the society and medical doctors. There are at least four different and well-accepted operational definitions of CFS (Holmes et al., 1988; Lloyd et al., 1990; Sharpe, 1991; Fukuda et al., 1994); however, all rely on the subjective reports, and there are no objective diagnostic discoveries. So it is generally difficult to define official CFS symptoms; however, it seems that many of them are similar as those reported by the patients with post-COVID-19 brain fog (Ross et al., 2004; Ocon, 2013; Paul et al., 2021; Theoharides et al., 2021; Wostyn, 2021; Asadi-Pooya et al., 2022; Callan et al., 2022; Hugon et al., 2022; Kopańska et al., 2022; Krishnan et al., 2022; Pierce et al., 2022; Premraj et al., 2022).

The etiology of brain fog in patients who coped with COVID-19 is still unknown. Some scientists postulate that SARS-CoV-2 that leads to infection also causes mitochondrial dysfunction (Pierce et al., 2022) in the brain, resulting in the viral genome and mitochondrial toxic interaction causing brain infection. The mitochondria are cellular structures that are crucial for understanding the pathophysiology and treatment of post-COVID-19 syndrome fatigue. Mitochondrial bioenergetic dysfunction may lead to anaerobic glycolysis to compensate for dysfunctional oxidative phosphorylation, as stated by Pierce (Pierce et al., 2022).

Brain cognitive functions are energy-sensitive and can be functionally altered or disrupted by the energy-requiring viral genome replication in the brain. The other mechanisms can also cause brain fog in COVID-19: inflammation, reduced tissue oxygenation, and reduced blood flow. This needs further validation at present (Pierce et al., 2022).

To the best of our knowledge, there was no systematic research involving investigations of brain cortical activity in the patients with post-COVID-19 brain fog symptoms using the dense array electroencephalography. We postulated that there may be a visible difference between the brain cortical activity, cognitive function disorders, and COVID-19, and that in future finding such a difference can help better understand the brain fog phenomena among those patients who got exposed, are recovering, and are coming back to their social activities.

In order to verify our hypotheses, we investigated a cohort of 120 patients which is divided into three sub-cohorts: those who suffered from COVID-19 and have symptoms of brain fog (sub-cohort A), suffered from COVID-19 and have no symptoms of brain fog (sub-cohort B), and the control group that had no COVID-19 and no symptoms (sub-cohort C).

We have selected participants for the sub-cohorts by surveying prospective participants and asking questions about their exposure to COVID-19, the time they suffered from the above-mentioned symptoms, and if they had at least three of them, we selected them for the sub-cohort A. If they had not any of the reported symptoms of brain fog and suffered from COVID-19 not earlier than 9 months before the EEG recordings, they were assigned for the sub-cohort B, and analogically if participants had neither reported COVID-19 nor symptoms of brain fog, they were assigned for the sub-cohort C. Assignment for the sub-cohorts A, B, and C were performed under the strict supervision of a medical doctor with experience in COVID-19 and post-COVID-19 patients' treatment.

Then we selected three commonly known tests with a large number of variants and modifications in experimental psychology that were supposed to be associated with typical symptoms of brain fog and were supposed to manifest some differences in the brain cortical activity. Those tests included: digit span (Lefebvre et al., 2005; Ostrosky-Soĺıs and Lozano, 2006; Leung et al., 2011; Woods et al., 2011), face recognition (Graham and Cabeza, 2001; Kaufmann et al., 2009; Wiese, 2012; Guillaume and Tiberghien, 2013), and task switching (Astle et al., 2006; Swainson et al., 2006; Fong et al., 2014; Gajewski et al., 2018).

The face recognition test is used to measure the ability to distinguish known faces from unknown ones, often without memory components, by asking participants whether they saw the displayed face before displaying new and shown-before faces one by another many times. The brain fog symptoms that were expected to manifest themselves during the face recognition experiment except the others: diminished visual and spatial skills, feelings of confusion and disorientation, and recognition of known faces in our opinion is associated with problems in finding names to objects.

The digit span test is used to measure working memory number storage capacity. The participants can see or hear a sequence of numerical digits and are asked to recall the sequence correctly, with increasingly longer sequences being tested in each trial. The brain fog symptoms that were expected to manifest themselves during the digit-span experiment were mainly: memory problems and inability to focus and concentrate.

The task-switching test is used to measure the ability to switch attention from one task to another by doing tasks A and B with being made to adapt quickly from one situation to another. The brain fog symptoms that were expected to manifest during task-switching experiment themselves were mainly as follows: troubles in problem-solving and having a hard time while calculating.

In our opinion, the selection of these tests was adequate and linked, in the above-mentioned way, with the brain fog syndrome, and we hoped to connect with the ERP signal information using them.

All participants of the research completed all three tests and implemented them in such a way that it was possible to calculate and plot ERP for each so-called cognitive electrode of the 256 electrode dense array EEG amplifier, which was used for the recordings.

In addition, the 5-min-long resting state signal was acquired. Using the avalanche technique described by Oren Shriki (Shriki et al., 2013) for feature extraction, the crucial electrodes were selected in the resting state signal and their activity was used to train the linear discriminant analysis (LDA) algorithms (Izenman, 2013).

The neural avalanches analysis identifies spatiotemporal cascades of neuronal activity in the data and is used to assess several aspects of cortical dynamics (Shriki et al., 2013). In particular, it is useful to characterize deviations in the balance of excitation and inhibition, and we were interested in probing the effect of COVID-19 on this balance and look for differences among the cohorts. Two features were extracted from the avalanche analysis: the branching parameter and the exponent of the avalanche size distribution.

Multiple neurological disorders are characterized by changes in the excitation–inhibition balance, e.g., autism and mild cognitive impairment (MCI). Thus, we were interested in checking whether the avalanche analysis exhibits differences among the cohorts because this could suggest possible interpretations. The avalanche analysis is non-linear and extracts information that does not exist in single-channel spectral features. Furthermore, using single-channel power-based features could easily result in a high-dimensional feature vector, which would in turn lead to overfitting in the classifier due to the limited number of datasets. In this sense, the avalanche analysis allowed us to extract a low-dimensional feature vector that characterizes the whole electrode array.

It will be proved that, it is possible to distinguish sub-cohorts based only on the resting state with promising and satisfactory LDA classifier efficiency.

The main contribution of this paper is showing the evidence of differences in the ERP shape among 3 group of subjects (A - the patients who suffered from COVID-19 and had brain fog symptom; B - suffered from COVID-19 and had no brain fog symptoms; C - were healthy and never COVID-19-ill) during solving three symptoms-finding-oriented tasks. It was also found in this study that it is possible to classify the resting state signal using neural avalanches analysis for feature extraction and linear discriminant analysis (LDA) (Izenman, 2013). After this first analysis, we can expect that there are permanent or long-lasting biomarkers in the EEG signal that are the characteristics of the brain fog phenomena. The relevance of this study can be also manifested in the idea of an EEG-based protocol of finding new, still unknown, cortical changes in the patients after the COVID-19 episode.

## 2. Materials and methods

For all those experiments, permission from the Bioethical Commission of Maria Curie-Sklodowska University in Lublin, Poland was granted.

### 2.1. Cohort recruitment

First, we recruited 120 participants, mainly among the students of Computer Science and Cognitive Science at Maria Curie-Sklodowska University in Lublin, along with the students there were some members of their families selected as well. There were 90 men and 30 women aged from 20 to 67, the average age was 24.88, and the standard deviation of 9.57.

The process of the recruitment under the supervision of an experienced medical doctor for COVID-19 treatment was already described in Introduction.

One should remember that the experimental cohort consisted of three sub-cohorts:

- A: A total of 40 post-COVID-19 subjects with serious symptoms of brain fog.
- B: A total of 40 post-COVID-19 subjects without symptoms of brain fog.
- C: A total of 40 healthy subjects who did not suffer from COVID-19, without any symptoms of the brain fog.

We have made some attempts to find out that brain fog was related to the COVID-19 infection and not to other diseases. The participants were asked to declare serious diseases like real CFS (present before COVID-19), cancer, and all other chronic diseases including mental disorders, and if they declared so, they were automatically excluded from the cohort that being built. In addition, we did our best to ensure that in the control cohort, there were no COVID-19 participants selected as they declared that neither they had ever COVID-19 symptoms, nor their family members, nor close relatives, and some of them had tests results.

### 2.2. EEG recordings

**Figure 1:**
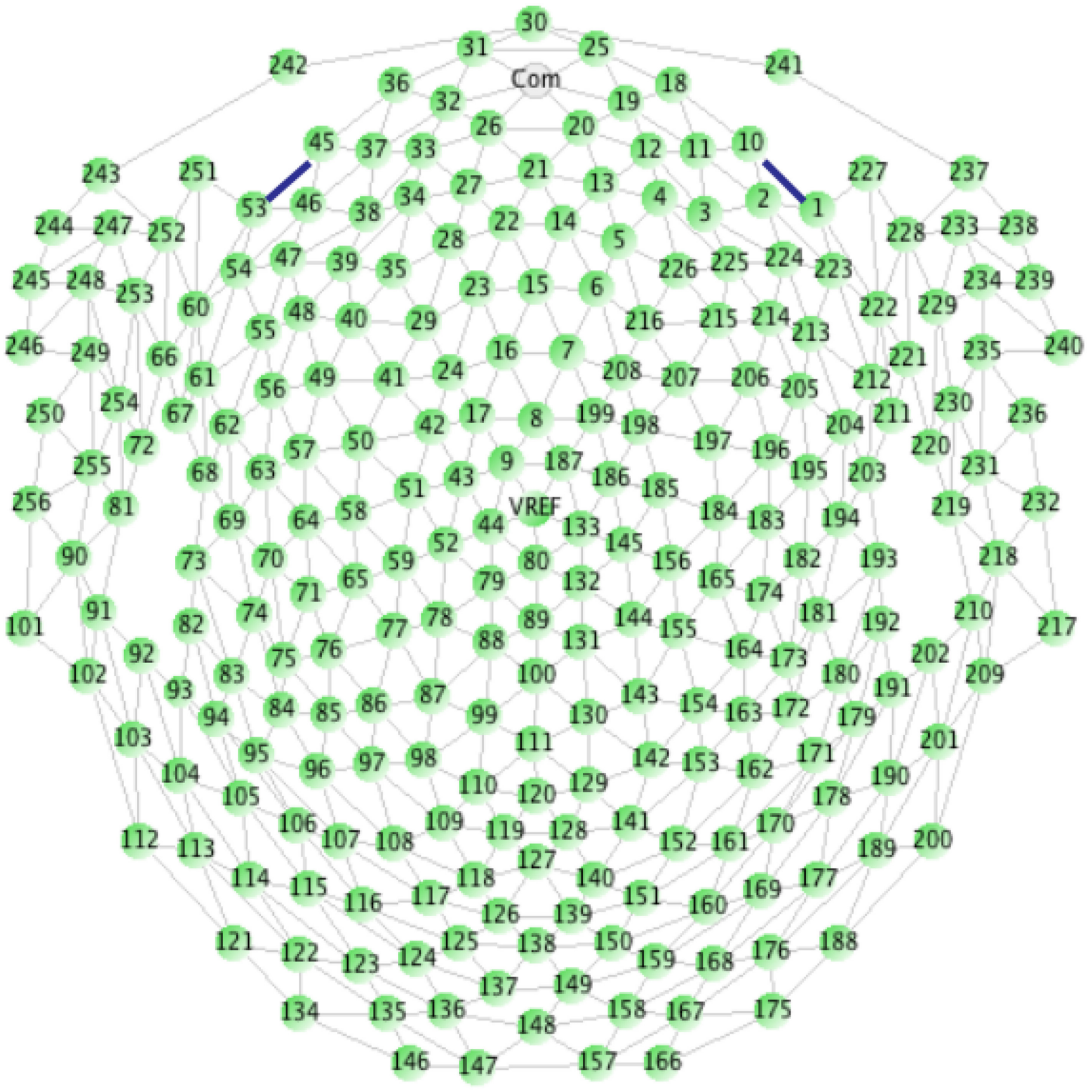
Electrodes placement on HydroCel GSN 130 Geodesic Sensor Net (Geodesics, 2007).

All EEG recordings were made using the 256-channel dens array EEG amplifier with HydroCel GSN 130 Geodesic Sensor Nets (see Figure 1) manufactured by Electrical Geodesic Systems (EGI)^1^ with 250 Hz sampling frequency. The amplifier works with the Net Station 4.5.4 software and SmartEye 5.9.7 for gaze calibration and eye-blinking or saccadic artifact removal. The lab is also equipped with geodesic photogrammetry system (GPS) working under control of the Net Local 1.00.00 and GeoSource 2.0. The ERP experiments are designed in the PST e-Prime 2.0.8.90.

### 2.3. ERP experiments

All subjects participated in three ERP, also mentioned in the Introduction, experiments on the same day in the sequence as follows:

#### 2.3.1. Face recognition

In this experiment, the participants were shown three faces to remember. They were presented on the screen for 6 s, one by one. Later there were repeated random sequences of 12 faces (including those to be remembered) and the participants were asked to click on the response pad each time they saw the known face. There were in total 270 trials in the experiment. The experiment continued until the whole sequence of faces was presented.

#### 2.3.2. Digit span

The participants were asked to remember what they heard in earphones and then recall from their memory by typing on the keyboard the sequences of numbers from 1 to 10. At the beginning, there were three-digit long sequences that increased in length by one up to 10-digit long sequences, after being repeated several times each time before the increase the length of the sequence by 1. In detail, the digits were presented as follows: 10 × 3-digit sequences, 10 × 4-digot, 5 × 5,6,7 an 8-digit sequences, 9 × 9-digit sequences, and 15 × 10-digit sequences. In one n-digit sequence series, there were always different sequences. There were in total 64 trials in this experiment. In some cases, the experiment was repeated from the beginning until 30 errors in the sequence recall were registered.

#### 2.3.3. Task switching

In the task-switching experiment, there was a computer screen divided into the top section and the bottom section. Each time there was a letter and a number shown either in the top or in the bottom section of the screen. The participants were asked to click button on the response pad if there was a vowel in the top section of the screen or if there was an even number in the bottom of the screen. There were in total 150 trials in this experiment. All participants declared that they knew what the vowel and even number were. The experiment continued until 75 vowels and 75 numbers were presented.

#### 2.3.4. Resting state

Then patients were asked to sit with closed eyes and do not move in order to record the 5 min of resting state signal.

After the resting state phase, the photograph using photogrammetry station was taken for the future source-localization experiment.

### 2.4. Preprocessing pipelines

#### 2.4.1. For ERP

**Figure 2:**
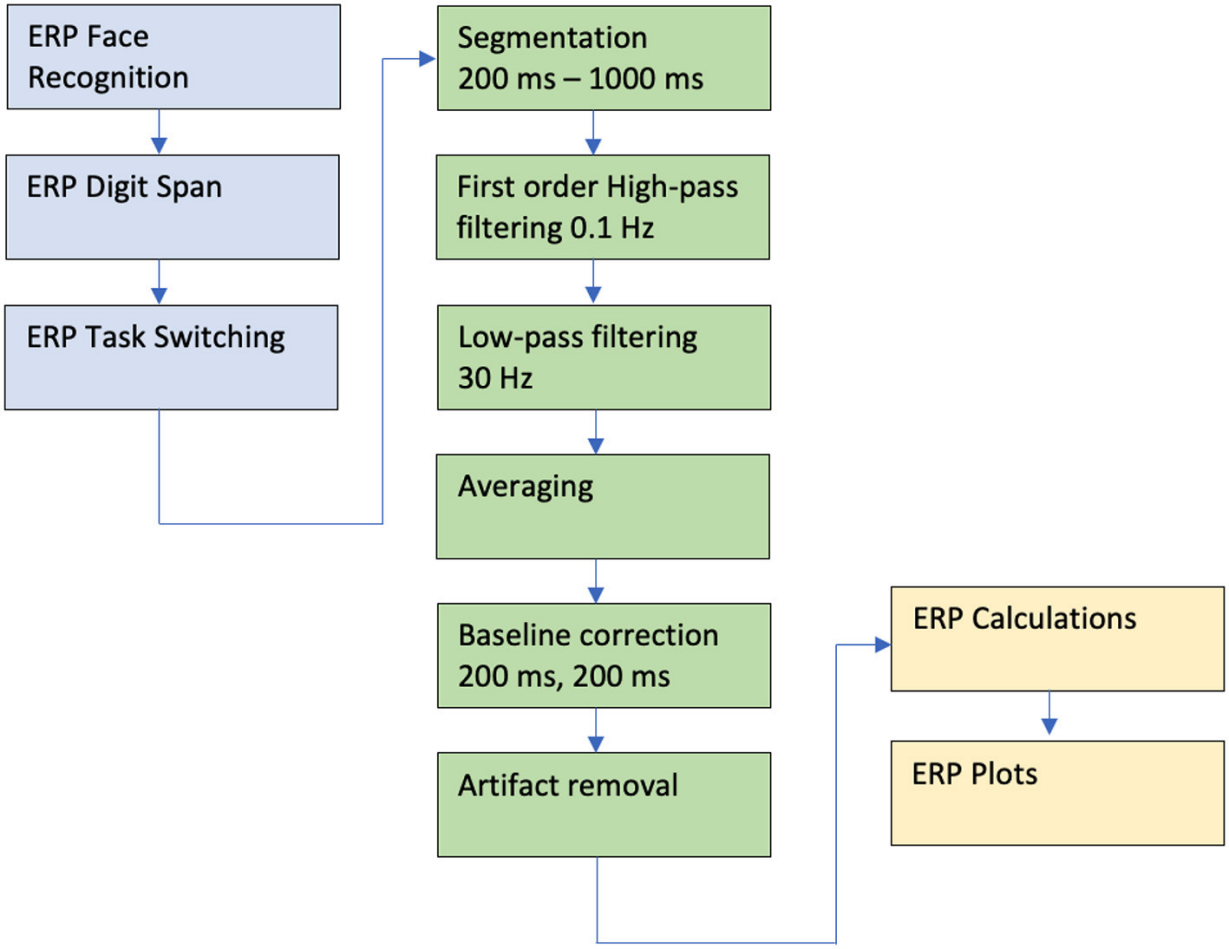
Data analysis pipeline for the ERP experiments. For detail see the text.

The collected signal was preprocessed using the following procedures and parameters set on the Net Station software: filtration with 0.1 Hz high-pass and 30 Hz low-pass filters; segmentation with parameters in the range of 200–1,000 ms; automatic and in some cases manual artifact removal; and averaging and baseline correction beginning at 200 ms and lasting for 200 ms (see Figure 2).

Our dense array amplifier recorded the ERP signal from all 256 electrodes. However, we expected to find differences on the so-called cognitive electrodes based on our previous experience in the cognitive processing EEG signal analysis (Kawiak et al., 2020; Kwasniewicz et al., 2021; Schneider et al., 2022). These electrodes are described in the EGI 256-channel cap specification as best for cognitive ERP observations, covering the scalp regularly, and numbered as follows: E98, E99, E100, E101, E108, E109, E110, E116, E117, E118, E119, E124, E125, E126, E127, E128, E129, E137, E138, E139, E140, E141, E149, E150, E151, and E152 (see Figure 1).

Even though the cohort consisted of 120 participants, its division into three sub-cohorts made it hard to receive smooth ERP curves. In such cases, a wide range of smoothing filters can be applied to different phases of data preparation, preprocessing, and cleaning (Kawala-Sterniuk et al., 2020). So, to generate better plots additionally, we manually removed the rest of the artifacts and the noise was reduced using polynomial smoothing.

We applied the grand average cross-correlation described in Luck (2005) for the ERP calculations and the R 4.1.3 version of the developer's statistical environment.

#### 2.4.2. For classification

All recordings went through the same preprocessing pipeline performed in Matlab, using the EEGLAB toolbox and the custom code. At first, the high-pass filter (1 Hz cutoff) was applied, and then non-scalp electrodes (radius > 0.5) leaving 137 channels were removed. The line noise (45–55 Hz) was removed using the CleanLine EEGLAB extension. Consequently, the ASR algorithm using clean_rawdata EEGLAB extension was carried out and down-sampled the data to 125 Hz (origin sampling rate is 250 Hz) and channels re-reference to mean was performed. We applied ICA and EEGLAB IC Labels procedures. The resulting ICs identified as muscles, eyes or heart activities as well as line and channel noise were removed. Finally, a low pass filter was applied (40 Hz).

#### 2.4.3. Features extraction for classification

Due to for the relatively large number of electrodes, we conducted the neural avalanches analysis (Shriki et al., 2013; Yu et al., 2013; Lombardi et al., 2021), narrating the cortical dynamic.

**Figure 3:**
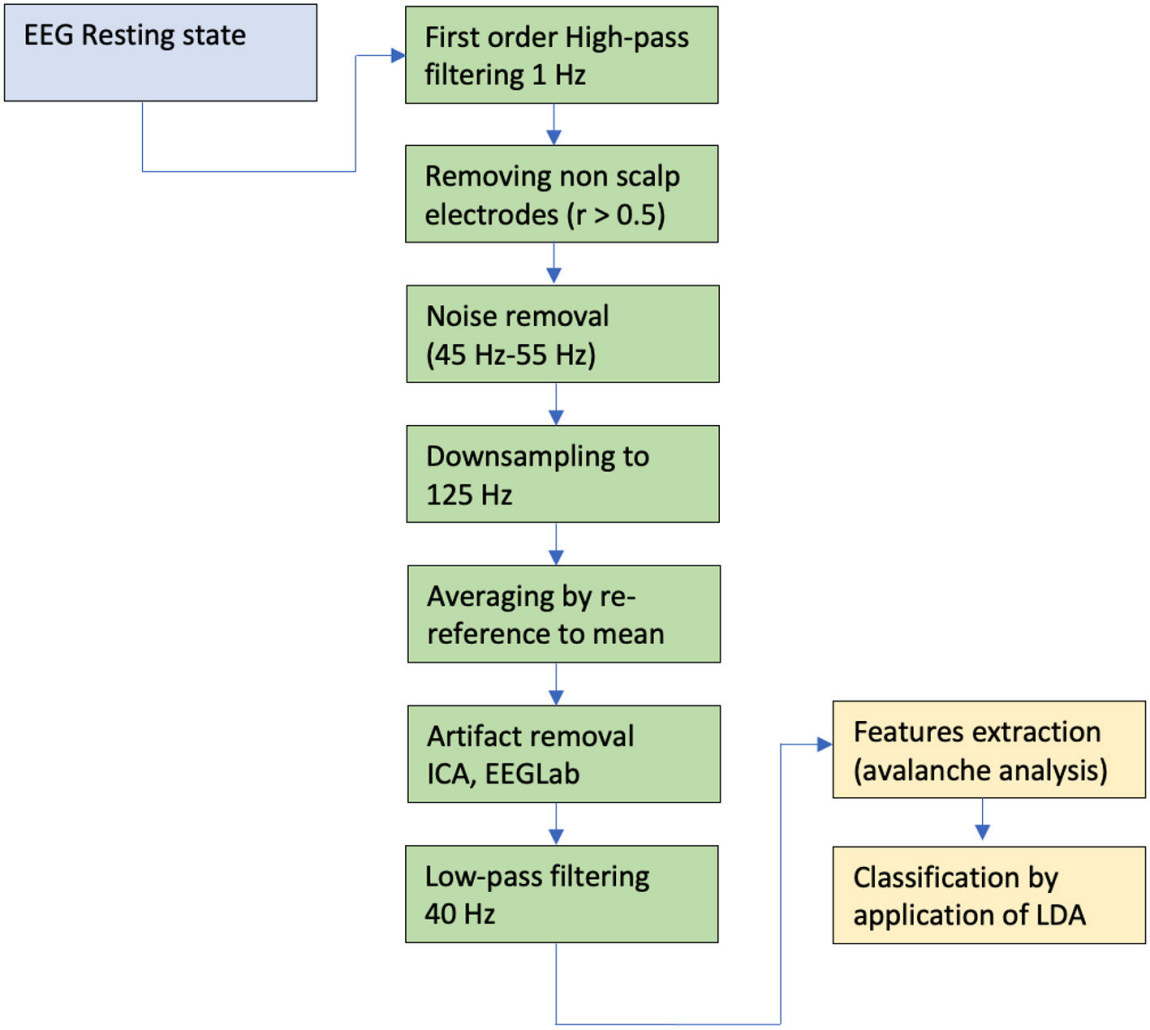
Data analysis pipeline for the resting state. For detail see the text.

The analysis followed in Arviv et al. (2015), with some adjustments, in relation to different methods, channels number and sampling rate. The SD thresholds were set to positive and negative 2.7. We applied the analysis to the C sub-cohort with 10 different time windows, Δt of 8 ms, and obtained the mean α and σ. We found that 3Δt of 24 ms yield agreeable results (α = –1.5336, *SD* = ±0.0620, σ = 1.1023, *SD* = ±0.1485, and *N* = 17). We then produced α and σ values for each recording using Δt = 3 (see Figure 3).

The linear discriminant analysis (LDA) (Izenman, 2013) was used for classification. Note that in Tables 1, 2, sensitivity is calculated as TP/P and specificity as TN/N, PPV = TP/(TP + FP), and NPV = TN/(TN + FN).

**Table 1:** Results of cross-validation.

	**Sensitivity**	**Std**	**Specifity**	**Std**	**PPV**	**Std**	**Fold size**
A & B vs. C	0.59621	0.093311	0.56944	0.34326	0.78671	0.16854	12
B & C vs. A	0.68889	0.30307	0.5849	0.091849	0.41587	0.18462	12
A vs. B	0.59286	0.29781	0.60408	0.25717	0.55119	0.17694	9
A vs. C	0.64444	0.27114	0.6	0.2708	0.60833	0.22454	7
B vs. C	0.55556	0.21465	0.81667	0.29107	0.79444	0.23134	8

**Table 2:** Results of classification performed using the test set.

	**Sensitivity**	**Specifity**	**PPV**	**NPV**
A & B vs. C	0.70833	0.75	0.89474	0.46154
B & C vs. A	0.63636	0.66667	0.5	0.77778
A vs. B	0.63636	0.53846	0.53846	0.63636
A vs. C	0.63636	0.875	0.875	0.63636
B vs. C	0.69231	0.75	0.81818	0.6

## 3. Results

### 3.1. ERP data analysis

#### 3.1.1. Event-related potential on cognitive electrodes

The first objective of the reported research was to examine whether there are differences in ERPs calculated for all three sub-cohorts.

**Figure 4:**
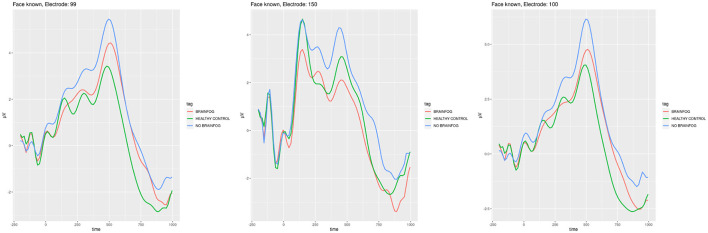
ERP plots generated for the electrodes **E99**, **E150**, and **E100** that showed smallest cross-correlation in the **face recognition experiment when the participants saw the known face**. Red line—the participants that suffered from COVID-19 and with brain fog symptoms. Blue line—the participants that suffered from COVID-19 and without brain fog symptoms. Green line—the participants without COVID-19 episode. See also Tables 3–5 in Appendix A.

**Figure 5:**
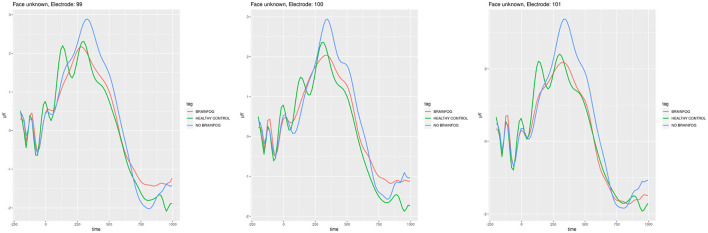
ERP plots generated for the electrodes **E99**, **E100**, and **E101** that showed the smallest cross-correlation in the **face recognition experiment when the participants saw the unknown face**. Red line—the participants that suffered from COVID-19 and with brain fog symptoms. Blue line—the participants that suffered from COVID-19 and without brain fog symptoms. Green line—the participants without COVID-19 episode. See also Tables 3–5 in Appendix A.

**Figure 6:**
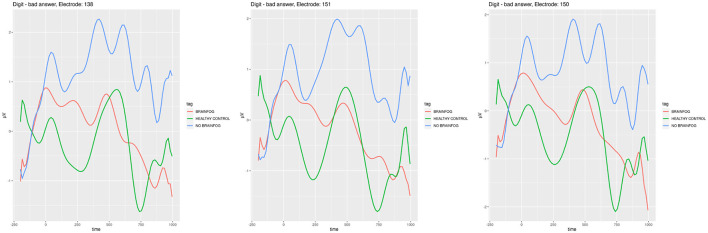
ERP plots generated for the electrodes **E138**, **E151**, and **E150** that showed the smallest cross-correlation in the **digit span experiment when the participants were wrong**. Red line—the participants that suffered from COVID-19 and with brain fog symptoms. Blue line—the participants that suffered from COVID-19 and without brain fog symptoms. Green line—the participants without COVID-19 episode. See also Tables 6–10 in Appendix A.

**Figure 7:**
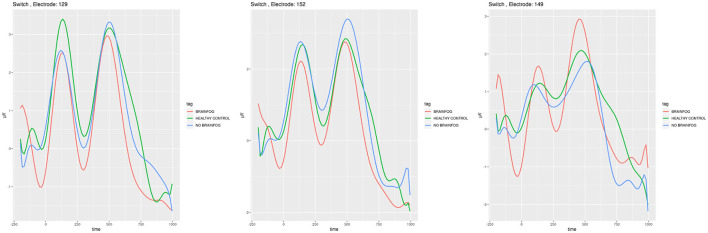
ERP plots generated for the electrodes **E129**, **E152**, and **E149** that showed the smallest cross-correlation in the **task-switching experiment when the participants saw digits**. Red line—the participants that suffered from COVID-19 and with brain fog symptoms. Blue line—the participants that suffered from COVID-19 and without brain fog symptoms. Green line—the participants without COVID-19 episode. See also Tables 11–13 in Appendix A.

**Figure 8:**
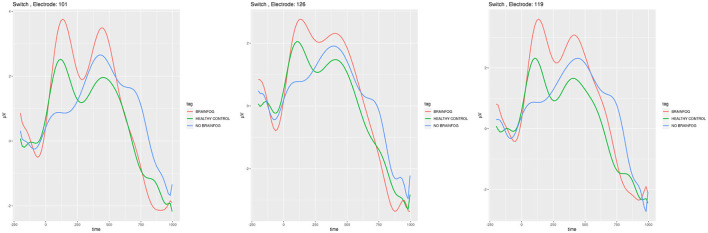
ERP plots generated for the electrodes **E101**, **E126**, and **E119** that showed the smallest cross-correlation in the **task-switching experiment when the participants saw letters**. Red line—the participants that suffered from COVID-19 and with brain fog symptoms. Blue line—the participants that suffered from COVID-19 and without brain fog symptoms. Green line—the participants without COVID-19 episode. See also Tables 14–17 in Appendix A.

We have used the cross-correlation to find out whether the signal in particular cases is not correlated. We found the differences that are presented in Figures 4–8.

In Figure 4, the ERPs of the least cross-correlated signal on the particular electrodes are presented for the face recognition test for the case when the participants saw the known faces, while in Figure 5, the case of unknown faces in the same experiment is shown.

In Figure 6, one can find ERPs calculated for the least correlated signal on the best manifesting electrodes in this case in the digit span experiment when the participants were wrong in recalling the sequences of numbers from the working memory.

In Figures 7, 8, the ERP analogical results are shown for the task-switching experiment for the digits and letters shown to the participants on the screen, respectively.

Tables 3–20 in Appendix A supporting the plots of Figures 4–8.

Note that the cross-correlation was applied to all cognitive electrodes; however, in Figures 4–8, the most interesting plots which are least correlated are presented.

One should also remember that the cross-correlation method is only one of many that can be applied to find the ERP differences in the particular sub-cohorts. The other one is, e.g., the difference in the amplitude that is clearly visible in the plots of Figures 4–8 even without calculations.

### 3.2. Machine-learning data analysis

The secondary objective of the research was to build machine-learning classifiers that model brain cortical dynamics of the cohort participants and classify this activity with high, >70%, accuracy. We are experienced in classifying the EEG cortical responses for the source localization data (Wojcik et al., 2018a,b, 2019a,b); however, for this research, we investigated the plain signal obtained from the amplifier. We have built five machine-learning LDA classifiers to process the collected resting state signal.

We have split the data into train and test data sets (70–30%) while maintaining all cohorts' proportions in both sets. We trained the five LDA classifiers for five different conditions. For each classifier, we performed the k-fold cross-validation evaluation, the “k” was set to 17%. We report the mean and standard deviations of sensitivity and specificity as well as positive predictive values, as shown in Table 1.

As the classifiers are binary, during the classification, we compared the following groups: (A&B vs. C), (B&C vs. A), (A vs. B), (A vs. C), and (B vs. C), where A is the group that recovered from COVID-19 with the brain fog symptoms, B is the group that recovered from COVID-19 without the brain fog symptoms, and C is the group that has not suffered from COVID-19 at all.

The classification results performed on the test set are very promising and range from 64 to 71% in distinguishing (C vs. A,B) 71%, (B,C vs. A) 64%, (A vs. B) 64%, (A vs. C) 64%, and (B vs. C) 69%. As one can see in Table 2, we can classify, with higher than 70% accuracy, the cases of the participants that suffered from COVID-19 and the healthy ones, and also a high degree of classification efficiency is achieved for distinguishing healthy patients from those that recovered from COVID-19 and have no brain fog symptoms. Moreover, now it is possible to create the voting agents system that include the both machine learning and ERP analysis, which can divide participants with perfect accuracy into all three groups based only on the results of the electrophysiological EEG data analysis.

## 4. Discussion

### 4.1. Event-related potentials

We hypothesized that there may be a difference in the cortical activity among the patients with brain fog, without brain fog, and healthy participants of the control group. Indeed, such a difference was found in the ERP signals using the cross-correlation method and the amplitude difference observation.

Of the three experiments, the highest cross-correlation between the ERP signal of all three sub-cohorts could be observed in the face recognition test (see Figures 4, 5 and Tables 13–10 in Appendix A). It is commonly accepted that with face recognition, N400 (James et al., 2001; Caldara et al., 2004; Balconi and Pozzoli, 2005), and N170 potentials in the spectrum of the ERP (Bentin and Deouell, 2000) are associated. During the experiment, the participants generally knew which face was to be remembered and which was new. Even though there is relatively the biggest correlation among all sub-cohorts, one can observe in Figures 4, 5 the difference in amplitudes. The sub-cohort who suffered with COVID-19 and the participants without brain fog had the highest amplitude. The lowest amplitude in general was characteristic of the members of a healthy control group, except for the electrode E150, which is positioned somewhere above in the right hemisphere occipital lobe visual cortex. One should note the local maximum in the amplitude near 170 ms and 400 ms, which in a way confirms the appropriate accomplishment of the experiment. The sequence of amplitudes requires future investigations; however, we could postulate that the healthy participants have least difficulties in solving the problem, while the brain fog sub-cohort oscillates somewhere between the regular COVID-19 and healthy control.

These amplitude differences and the sequence similar to that discussed earlier are the most visible during the digit span experiment (see Figure 6 and Tables 11–13 in Appendix A). In fact, in the digit span test, the cross-correlations were the smallest. In the literature, the N200 potential is associated with auditory discrimination that could play a role in the digit span experiment (Lim et al., 1999). Also, P200 and P300 are associated with the working memory condition as presented in Polich et al. (1983), Lefebvre et al. (2005), and Dong et al. (2015) where the digit span was also used for research. In Figure 6, a clear, notable difference in the ERP shape can be observed near 200 ms. Moreover, the gradient and general signal tendencies are completely different in the healthy control than in those with COVID-19 brain fog and no brain fog sub-cohorts. In the healthy control, one can note the sharp minimum, while among those who suffered from COVID-19, there are slowly decreasing waves. Again, the brain fog sub-cohort is placed somewhere between the healthy control and the regular COVID-19 sub-cohorts plots.

The complete mish-mash among the ERP plots, amplitude sequences, and cross-correlations can be observed after the task-switching experiment (see Figures 7, 8 and Tables 14–20 in Appendix A). The ERP investigations for the task-switching experiment were extensively used in this research, in fMRI, and in the dense sensors nets (Swainson et al., 2003; Astle et al., 2008; Mueller et al., 2009; Gajewski et al., 2018) with a similar variety of findings. In order to put in the logical order, our results should consider the task-switching experiment in its two sub-variants: the first when the participants decided about the digit (see Figure 7 and Tables 14–17 in Appendix A) and the second when the participants decided about the letter (see Tables 18–20 in Appendix A).

In the case when the participants could see digits (see Figure 7 and Tables 14–17 in Appendix A), there is a similar tendency observable in the ERP plots with a minima of around 200 ms and 500 ms. The 200 ms is reported in Scisco et al. (2008) where the authors use the task-switching experiment for the cardiovascular fitness investigations. The 500 ms time is also reported in Karayanidis et al. (2011) and is related to the pre-target negativity. The differences in amplitude can be observed on particular electrodes and as the cross-correlation method proves—they are not correlated.

In the case when the participants could see letters (see Figure 8 and Tables 18–20 in Appendix A), there is a distinct maximum observable for the regular COVID-19 in all three sub-cohorts in around 350–400 ms. It is stated in Jackson et al. (2004) that they are associated with the language skills, so in a way with letters and our results also confirm the appropriate set up of the experiment. The sequence of amplitudes is the same, the highest for brain foggy sub-cohort A, medium for sub-cohort B, and the lowest for healthy sub-cohort C. Nevertheless, the obvious amplitude and cross-correlation difference in the task-switching experiment verify our hypotheses positively and similar to the two other experiments achieve the primary objective of this study.

One should note that in Figures 4–8 and Tables 3–20 in Appendix A, only the data for the least cross-correlated electrodes are presented. However, the cross-correlation analysis confirmed our expectations and followed our findings on all cognitive electrodes. This justifies our choice of the ERP experiment to be conducted during the project realization.

In addition, our approach can be an interesting idea for designing a new EEG-based test for the COVID-19 episodes. Such a test can be useful especially when we find someone who reports being healthy and not suffering from COVID-19 before, however, possessing cortical activity characteristics of the target group.

### 4.2. Resting state classification

Before addressing the machine-learning results, we should consider the relatively small amount of data (yet of high quality) that was included in the study. Also, most of the classifiers dealt with highly imbalanced classes.

Most of the classifiers showed better performance in the metrics reported on the test set than in the k-fold cross-validation. This could be explained by the larger amount of data available in the test set than in the train set; hence, each mistake had a smaller significance.

The A&B vs. C classifier had sensitivity of 0.70833, while the COVID-19 samples were 75% of the cases, so it underperformed a null classifier (appointing the “1” label to all samples). Yet the specificity indicates that 6 out 8 samples were identified correctly as C (healthy, no COVID-19) cases.

The B&C vs. A classifier had consistently poor results in validation and test performances. This could be attributed to the fact that we grouped the B and C groups that have reasonably claimed to be essentially different (COVID-19 with no brain fog symptoms and healthy, no COVID-19). This result could also indicate that the brain fog effect can not be identified in the domain of neural dynamics.

The another support for this claim are the A versu B classifier results that did not rise above the chance level.

The A vs. C and B vs. C classifiers results are similar. The sensitivity was slightly higher than that of null classifiers (0.5789 and 0.61904) but not by a large margin. Yet the specificity reveals that most of the healthy subjects of group C were accurately identified. The positive predictive value and the negative predictive value indicate that the mistake tends to be the wrong classification of B cases as C.

Although the topic might be quite different from that of the present study, the methodology, that is the use of machine-learning techniques for automatic identification and classification of symptoms is similar to that of other studies (Dipaola et al., 2019, 2021).

### 4.3. Future research

In the post-COVID-19 era, there is a wide variety of problems to solve in all areas of the global economy. The mental health of society will be one of the most important issues to address in the scientific effort. The main challenge of the reported research was to find differences in the ERP and resting state signal possible to be distinguished either by the cross-correlation or amplitude analysis or using classifiers.

Having developed models and classifiers as mentioned in Tadeusiewicz (2015) and Salankar et al. (2022), in future it may be possible to predict the probability of brain fog episodes in patients that have recovered < 2 months before the first brain fog symptoms, which can help doctors to prepare better therapy and brain fog prevention paths. Such an approach requires, however, additional self-arranged tests carried out by volunteers for finding COVID-19 antibodies and are not predicted to be obligatory for this research. In the next step, this can lead to a wide range of innovations and a new kind of therapies researched, invented, and introduced in the post-pandemic reality, including the Industry 4.0 (Rojek et al., 2020a) approach and 3D printing of therapeutic toys or tools (Rojek et al., 2020b) to increase the comfort of life in future patients, thus making the research presented herein justified.

This is the first article in the series planned to be published in connection with the greater project realization. In the future, we will apply the source localization techniques in order to find differences in the geodesic activity of the cortex between sub-cohorts. As this is only the initial stage of the research, we plan and hope to get better insight into the brain fog phenomena providing a better understanding that will result in a better diagnosis and therapy planning toward faster and full recovery as future pandemics are predicted to come in the next decades.

Global warming and climate change together with the decreasing number of species and intensive migrations support the development of new viruses and some of them probably will require future lockdowns and similar COVID-19 commonly known restrictions. That is why the outcome of the research was our motivation for finding a methodology that will allow predicting brain fog proneness on time, thus improving the comfort of the life of future patients.

## Data availability statement

The raw data supporting the conclusions of this manuscript will be made available by the authors, without undue reservation to any qualified researcher.

## Ethics statement

The studies involving human participants were reviewed and approved by the Maria Curie-Sklodowska University's Bioethical Commission (MCSU Bioethical Commission permission 9.07.2021). The patients/participants provided their written informed consent to participate in this study. Written informed consent was obtained from the individual(s) for the publication of any potentially identifiable images or data included in this article.

## Author contributions

GW: head of the project, research idea and coordination, data science pipeline design, idea of the minimal cross-correlation finding, manuscript writing, literature review, and project fundraising. OS: head of the research carried out by the partner institution and data science pipeline design. LK: ERP data analysis and ERP plots smoothing and manual artifact removal. AK: ERP data science analysis and psychology. YB-H: cross-validation and machine-learning EEG resting state signal. SF: code for avalanche analysis. KW: EEG recordings, psychological surveying, and work in the laboratory. BB: EEG recordings, psychological surveying, and work in the laboratory. EP: project fundraising, recruitment of participants, surveying, literature review, project reporting, and coordination support. All authors contributed to the article and approved the submitted version.

## Footnotes

^1^500 East 4th Ave. Suite 100, Eugene, OR 97401, USA.
