# Supplementary material for: Investigating brain cortical activity in patients with post-COVID-19 brain fog
Source: Front Neurosci. 2023 Feb 9;17:1019778. doi: 10.3389/fnins.2023.1019778 (PMC9947499; doi:10.3389/fnins.2023.1019778)
Supplement: Supplementary file 1 [file Table_1.pdf]

1 Appendix A - Supplementary material for "Investigating brain cortical activity in patients with post-COVID-19 brain fog."

	Lag	CCF
1	-10.00	0.44
2	-9.00	0.48
3	-8.00	0.52
4	-7.00	0.56
5	-6.00	0.60
6	-5.00	0.65
7	-4.00	0.69
8	-3.00	0.73
9	-2.00	0.77
10	-1.00	0.81
11	0.00	0.84
12	1.00	0.84
13	2.00	0.83
14	3.00	0.82
15	4.00	0.81
16	5.00	0.80
17	6.00	0.78
18	7.00	0.77
19	8.00	0.75
20	9.00	0.73
21	10.00	0.72

Table 1: Face Recognition - known faces. Electrode E99. Cross-Correlation results between the sub-cohorts A and C. Lag length 4 ms.

	Lag	CCF
1	-10.00	0.51
2	-9.00	0.55
3	-8.00	0.60
4	-7.00	0.66
5	-6.00	0.71
6	-5.00	0.77
7	-4.00	0.82
8	-3.00	0.87
9	-2.00	0.91
10	-1.00	0.94
11	0.00	0.96
12	1.00	0.94
13	2.00	0.91
14	3.00	0.87
15	4.00	0.82
16	5.00	0.77
17	6.00	0.71
18	7.00	0.65
19	8.00	0.59
20	9.00	0.54
21	10.00	0.48

Table 2: Face Recognition - known faces. Electrode E150. Cross-Correlation results between the sub-cohorts A and B. Lag length 4 ms.

	Lag	CCF
1	-10.00	0.78
2	-9.00	0.79
3	-8.00	0.80
4	-7.00	0.81
5	-6.00	0.82
6	-5.00	0.83
7	-4.00	0.83
8	-3.00	0.84
9	-2.00	0.84
10	-1.00	0.84
11	0.00	0.84
12	1.00	0.82
13	2.00	0.79
14	3.00	0.76
15	4.00	0.73
16	5.00	0.70
17	6.00	0.67
18	7.00	0.64
19	8.00	0.61
20	9.00	0.58
21	10.00	0.55

Table 3: Face Recognition - known faces. Electrode E100. Cross-Correlation results between the subcohorts B and C. Lag length 4 ms.

	Lag	CCF
1	-10.00	0.70
2	-9.00	0.71
3	-8.00	0.73
4	-7.00	0.75
5	-6.00	0.77
6	-5.00	0.80
7	-4.00	0.82
8	-3.00	0.84
9	-2.00	0.87
10	-1.00	0.89
11	0.00	0.91
12	1.00	0.89
13	2.00	0.87
14	3.00	0.85
15	4.00	0.83
16	5.00	0.81
17	6.00	0.79
18	7.00	0.77
19	8.00	0.75
20	9.00	0.73
21	10.00	0.71

Table 4: Face Recognition - unknown faces. Electrode E100. Cross-Correlation results between the subcohorts A and C. Lag length 4 ms.

	Lag	CCF
1	-10.00	0.83
2	-9.00	0.84
3	-8.00	0.85
4	-7.00	0.86
5	-6.00	0.87
6	-5.00	0.89
7	-4.00	0.90
8	-3.00	0.91
9	-2.00	0.93
10	-1.00	0.94
11	0.00	0.95
12	1.00	0.93
13	2.00	0.92
14	3.00	0.90
15	4.00	0.87
16	5.00	0.85
17	6.00	0.82
18	7.00	0.79
19	8.00	0.76
20	9.00	0.73
21	10.00	0.70

Table 5: Face Recognition - unknown faces. Electrode E100. Cross-Correlation results between the subcohorts A and B. Lag length 4 ms.

	Lag	CCF
1	-10.00	0.69
2	-9.00	0.70
3	-8.00	0.71
4	-7.00	0.72
5	-6.00	0.73
6	-5.00	0.74
7	-4.00	0.75
8	-3.00	0.76
9	-2.00	0.78
10	-1.00	0.80
11	0.00	0.82
12	1.00	0.80
13	2.00	0.78
14	3.00	0.76
15	4.00	0.73
16	5.00	0.70
17	6.00	0.68
18	7.00	0.65
19	8.00	0.62
20	9.00	0.60
21	10.00	0.57

Table 6: Face Recognition - unknown faces. Electrode E100. Cross-Correlation results between the subcohorts B and C. Lag length 4 ms.

	Lag	CCF
1	-10.00	0.67
2	-9.00	0.69
3	-8.00	0.71
4	-7.00	0.73
5	-6.00	0.76
6	-5.00	0.79
7	-4.00	0.81
8	-3.00	0.84
9	-2.00	0.87
10	-1.00	0.89
11	0.00	0.91
12	1.00	0.89
13	2.00	0.87
14	3.00	0.86
15	4.00	0.84
16	5.00	0.83
17	6.00	0.81
18	7.00	0.79
19	8.00	0.77
20	9.00	0.75
21	10.00	0.73

Table 7: Face Recognition - unknown faces. Electrode E99. Cross-Correlation results between the subcohorts A and C. Lag length 4 ms.

	Lag	CCF
1	-10.00	0.82
2	-9.00	0.83
3	-8.00	0.84
4	-7.00	0.86
5	-6.00	0.87
6	-5.00	0.89
7	-4.00	0.90
8	-3.00	0.92
9	-2.00	0.93
10	-1.00	0.94
11	0.00	0.95
12	1.00	0.94
13	2.00	0.92
14	3.00	0.90
15	4.00	0.87
16	5.00	0.84
17	6.00	0.81
18	7.00	0.78
19	8.00	0.75
20	9.00	0.72
21	10.00	0.69

Table 8: Face Recognition - unknown faces. Electrode E101. Cross-Correlation results between the subcohorts A and B. Lag length 4 ms.

	Lag	CCF
1	-10.00	-0.28
2	-9.00	-0.30
3	-8.00	-0.31
4	-7.00	-0.33
5	-6.00	-0.35
6	-5.00	-0.36
7	-4.00	-0.38
8	-3.00	-0.39
9	-2.00	-0.41
10	-1.00	-0.42
11	0.00	-0.43
12	1.00	-0.42
13	2.00	-0.42
14	3.00	-0.42
15	4.00	-0.42
16	5.00	-0.42
17	6.00	-0.42
18	7.00	-0.42
19	8.00	-0.42
20	9.00	-0.42
21	10.00	-0.42

Table 9: Digit Span - wrong answers. Electrode E138. Cross-Correlation results between the sub-cohorts A and C. Lag length 4 ms.

	Lag	CCF
1	-10.00	0.31
2	-9.00	0.31
3	-8.00	0.30
4	-7.00	0.30
5	-6.00	0.29
6	-5.00	0.29
7	-4.00	0.28
8	-3.00	0.28
9	-2.00	0.27
10	-1.00	0.27
11	0.00	0.26
12	1.00	0.24
13	2.00	0.22
14	3.00	0.19
15	4.00	0.17
16	5.00	0.14
17	6.00	0.11
18	7.00	0.09
19	8.00	0.07
20	9.00	0.05
21	10.00	0.02

Table 10: Digit Span - wrong answers. Electrode E151. Cross-Correlation results between the sub-cohorts A and B. Lag length 4 ms.

	Lag	CCF
1	-10.00	-0.61
2	-9.00	-0.62
3	-8.00	-0.62
4	-7.00	-0.63
5	-6.00	-0.64
6	-5.00	-0.65
7	-4.00	-0.66
8	-3.00	-0.67
9	-2.00	-0.68
10	-1.00	-0.69
11	0.00	-0.71
12	1.00	-0.70
13	2.00	-0.69
14	3.00	-0.68
15	4.00	-0.67
16	5.00	-0.66
17	6.00	-0.65
18	7.00	-0.63
19	8.00	-0.62
20	9.00	-0.62
21	10.00	-0.61

Table 11: Digit Span - wrong answers. Electrode E150. Cross-Correlation results between the sub-cohorts B and C. Lag length 4 ms.

	Lag	CCF
1	-10.00	0.10
2	-9.00	0.15
3	-8.00	0.20
4	-7.00	0.25
5	-6.00	0.30
6	-5.00	0.34
7	-4.00	0.37
8	-3.00	0.41
9	-2.00	0.43
10	-1.00	0.46
11	0.00	0.48
12	1.00	0.50
13	2.00	0.52
14	3.00	0.53
15	4.00	0.54
16	5.00	0.54
17	6.00	0.54
18	7.00	0.53
19	8.00	0.52
20	9.00	0.50
21	10.00	0.49

Table 12: Task Switching - digits shown. Electrode E129. Cross-Correlation results between the sub-cohorts A and C. Lag length 4 ms.

	Lag	CCF
1	-10.00	-0.00
2	-9.00	0.04
3	-8.00	0.09
4	-7.00	0.13
5	-6.00	0.18
6	-5.00	0.22
7	-4.00	0.26
8	-3.00	0.30
9	-2.00	0.34
10	-1.00	0.38
11	0.00	0.41
12	1.00	0.43
13	2.00	0.45
14	3.00	0.47
15	4.00	0.48
16	5.00	0.49
17	6.00	0.49
18	7.00	0.48
19	8.00	0.48
20	9.00	0.47
21	10.00	0.46

Table 13: Task Switching - digits shown. Electrode E129. Cross-Correlation results between the sub-cohorts A and B. Lag length 4 ms.

	Lag	CCF
1	-10.00	0.18
2	-9.00	0.23
3	-8.00	0.28
4	-7.00	0.33
5	-6.00	0.38
6	-5.00	0.42
7	-4.00	0.46
8	-3.00	0.50
9	-2.00	0.53
10	-1.00	0.55
11	0.00	0.56
12	1.00	0.56
13	2.00	0.56
14	3.00	0.54
15	4.00	0.52
16	5.00	0.50
17	6.00	0.46
18	7.00	0.43
19	8.00	0.39
20	9.00	0.35
21	10.00	0.31

Table 14: Task Switching - digits shown. Electrode E152. Cross-Correlation results between the sub-cohorts A and B. Lag length 4 ms.

	Lag	CCF
1	-10.00	0.56
2	-9.00	0.59
3	-8.00	0.62
4	-7.00	0.65
5	-6.00	0.67
6	-5.00	0.70
7	-4.00	0.72
8	-3.00	0.75
9	-2.00	0.76
10	-1.00	0.77
11	0.00	0.78
12	1.00	0.78
13	2.00	0.77
14	3.00	0.76
15	4.00	0.74
16	5.00	0.73
17	6.00	0.71
18	7.00	0.68
19	8.00	0.66
20	9.00	0.64
21	10.00	0.62

Table 15: Task Switching - digits shown. Electrode E149. Cross-Correlation results between the sub-cohorts B and C. Lag length 4 ms.

	Lag	ACF
1	-10.00	0.85
2	-9.00	0.86
3	-8.00	0.87
4	-7.00	0.89
5	-6.00	0.90
6	-5.00	0.91
7	-4.00	0.92
8	-3.00	0.93
9	-2.00	0.94
10	-1.00	0.95
11	0.00	0.95
12	1.00	0.95
13	2.00	0.95
14	3.00	0.94
15	4.00	0.93
16	5.00	0.92
17	6.00	0.90
18	7.00	0.89
19	8.00	0.88
20	9.00	0.87
21	10.00	0.86

Table 16: Task Switching - letters shown. Electrode E101. Cross-Correlation results between the sub-cohorts A and C. Lag length 4 ms.

	Lag	ACF
1	-10.00	0.84
2	-9.00	0.84
3	-8.00	0.85
4	-7.00	0.86
5	-6.00	0.87
6	-5.00	0.88
7	-4.00	0.88
8	-3.00	0.89
9	-2.00	0.90
10	-1.00	0.90
11	0.00	0.91
12	1.00	0.90
13	2.00	0.88
14	3.00	0.87
15	4.00	0.85
16	5.00	0.84
17	6.00	0.82
18	7.00	0.80
19	8.00	0.78
20	9.00	0.77
21	10.00	0.75

Table 17: Task Switching - letters shown. Electrode E126. Cross-Correlation results between the sub-cohorts A and B. Lag length 4 ms.

	Lag	ACF
1	-10.00	0.71
2	-9.00	0.71
3	-8.00	0.71
4	-7.00	0.72
5	-6.00	0.73
6	-5.00	0.74
7	-4.00	0.75
8	-3.00	0.75
9	-2.00	0.76
10	-1.00	0.76
11	0.00	0.76
12	1.00	0.74
13	2.00	0.73
14	3.00	0.71
15	4.00	0.69
16	5.00	0.67
17	6.00	0.65
18	7.00	0.63
19	8.00	0.61
20	9.00	0.59
21	10.00	0.57

Table 18: Task Switching - letters shown. Electrode E137. Cross-Correlation results between the sub-cohorts B and C. Lag length 4 ms.
